# Anisotropy in the Tensile Properties of a Selective Laser Melted Ti-5Al-5Mo-5V-1Cr-1Fe Alloy during Aging Treatment

**DOI:** 10.3390/ma15165493

**Published:** 2022-08-10

**Authors:** Hualong Huang, Taomei Zhang, Chao Chen, Seyed Reza Elmi Hosseini, Jiaqi Zhang, Kechao Zhou

**Affiliations:** 1 State Key Laboratory of Powder Metallurgy, Powder Metallurgy Research Institute, Central South University, Changsha 410083, China; 2 School of Metallurgy and Materials Engineering, Iran University of Science and Technology (IUST), Narmak, Tehran 13114-16846, Iran

**Keywords:** selective laser melting, Ti-5Al-5Mo-5V-1Cr-1Fe, mechanical anisotropy, GND density

## Abstract

In this work, the anisotropic microstructure and mechanical properties of selective laser melted (SLMed) Ti-5Al-5Mo-5V-1Cr-1Fe (Ti-55511) alloy before and after aging treatment are investigated. Owing to the unique thermal gradient, the prior columnar β grains with {001} texture component grow in the building direction, and the mechanical properties of the as-fabricated Ti-55511 alloy exhibit slight anisotropy. Aging treatment creates uniform precipitation of the α phase at the boundaries as well as the interior of β grains. Due to the microstructure of the aged samples with a weak texture, the mechanical properties exhibit almost isotropic characteristics with an ultimate tensile strength of 1133 to 1166 MPa, yield strength of 1093 to 1123 MPa, and elongation from 13 to 16%, which meet the aerospace allowable specification very well. By XRD and EBSD analyses, the total dislocation density of the aged samples (~134.8 × 10^13^ m^−2^) is significantly lower than that of the as-fabricated samples (~259.4 × 10^13^ m^−2^); however, the aged samples exhibit a higher geometrically necessary dislocation (GND) density (~28.5 × 10^13^ m^−2^) compared with the as-fabricated samples GND density (~2.9 × 10^13^ m^−2^). Thus, a new approach to strengthening theory for estimating the anisotropic mechanical properties of AM alloys is proposed.

## 1. Introduction

Ti-5Al-5Mo-5V-1Cr-1Fe (Ti-55511) is a typical near β titanium alloy, which has been widely used in aircraft and aerospace components due to its high strength to weight ratio, excellent hardenability, and corrosion resistance as well as superior mechanical properties. Furthermore, the high cost of design and manufacturing of such structural components has prompted interest in the possibility of using additive manufacturing (AM) techniques, which mainly include the powder bed fusion (PBF) and directed energy deposition (DED) processes [1]. The techniques that could be suggested in PBF are selective laser melting (SLM), electron beam melting (EBM), selective laser sintering (SLS), direct metal laser sintering (DMLS), laser metal fusion (LMF), etc. Moreover, the techniques that could be supposed in DED are laser metal deposition (LMD), direct manufacturing (DM), direct metal deposition (DMD), laser-engineered net shaping (LENS), wire arc additive manufacturing (WAAM), etc. There is significant microstructural anisotropy in AM components. This is mainly derived from the layer-by-layer nature of the AM process, which introduces a unique thermal history at any location with the combined effects of rapid solidification and cyclic heat treatment.

Numerous researchers have focused on the relationship between the anisotropic microstructure and the mechanical properties. For instance, Hrabe et al. showed that the distance of the laser source from built plate, part size, and orientation of the XY-plane have a slight effect on the microstructure and mechanical properties of EBMed Ti-6Al-4V alloy [2,3]. Liu et al. suggested that columnar grain morphology and crystallographic texture are the main factors causing anisotropic behaviors [4,5,6,7]. Carroll et al. found that the anisotropy in ductility of DEDed Ti-6Al-4V is attributed to the columnar prior β grains morphology as well as the presence of the grain boundary α, which serves as a path along which damage can preferentially accumulate, leading to fracture [8]. It has been demonstrated that a strong β<100> texture component along the building direction was formed during the fabricating process of the Ti-6Al-4V alloy made by EBM and the electron beam rapid manufacturing (EBRM) techniques. However, the β textures display mainly a simple (110)//building direction of the fiber texture component of SLMed Ti-13Nb-13Zr alloy [9]. Upon cooling, the fiber texture component would transform to the α texture component based on the Burgers orientation relationship (BOR), that is, {0001}_α_//{110}_β_ and <1120>_α_//<111>_β_. Such a transformed α texture plays a significant role in determining the anisotropic tensile properties, which leads to a higher tensile strength for the sample loaded along the 45° direction than that of the vertical and horizontal directions [10,11].

To further understand the regularity, many researchers have tried to establish models to explain and verify the experimental results. Carroll et al. showed that the grain boundary α could be subjected to the Mode I of the opening failure when applying tension along the horizontal direction. However, the grain boundary α is not subjected to the Mode I of opening failure when applying tension along the vertical direction [8]. Zhou et al. reported that the horizontal and vertical samples undergo different deformation processes before the transcrystalline fracture, and the prior-β grains in the vertical sample can resist more against the damage than in the horizontal sample. Moreover, cracks are more likely to appear in the horizontal sample under a tensile opening mode, which could fail eventually [9]. Wang et al. suggested that the number of grain boundaries encountered in dislocation motion could be calculated by only considering the direction of the maximum shear stress (at an angle of 45° to the direction of stretching) rather than the direction of the maximum normal stress (stretching direction) [7]. Hicks et al. showed that microvoids which were created along the β grain boundaries facilitated the crack propagation perpendicular to the loading direction when the columnar β grains were oriented perpendicular to the loading direction (i.e., horizontal specimens) [12]. Liu et al. indicated that the effective slip path for a sample loaded along the vertical direction is approximately equal to that in the horizontal direction. In the meantime, the effective slip path for the sample loaded along the 45° direction is obviously longer than that of the two directions above [4].

Despite the widely studied α + β alloys such as the Ti-6Al-4V alloy [13,14,15,16,17,18], AM of near β-titanium alloys such as the Ti-55511 alloy has attracted less attention. There are some reports on the microstructure, crystallographic texture, dislocation density, and mechanical properties of the AMed Ti-55511 alloy, which are demonstrated in Table 1 [19,20,21,22,23,24,25,26,27,28]. As is shown in this table, the AMed Ti-55511 alloy exhibits obvious anisotropy in mechanical properties both before and after the heat treatment. Moreover, The Ti-55511 alloy contains higher β stabilizing elements which stabilize more retained β phase in equilibrium conditions than the Ti-6Al-4V alloy [29]. Previous studies have shown the influence of process parameters and direct aging treatments on the microstructure and mechanical properties of SLMed Ti-55511 alloy [25,28]. Cui et al. have exhibited that the geometrically necessary dislocations (GNDs) are heterogeneously distributed and play an important role in the strengthening of AMed alloy [30,31]. These would imply that the microstructure, crystallographic texture, and dislocation density may change the mechanical properties of SLMed Ti-55511 alloy. The anisotropy in the microstructure and mechanical properties of SLMed Ti-55511 alloy is still not well understood. Thus, the microstructure, crystallographic texture, and dislocations behavior in SLMed Ti-55511 alloy before and after direct aging were studied in the present work. Moreover, the tensile properties of the Ti-55511 alloy before and after direct aging were tested in six typical orientations. Furthermore, the effects of microstructure, crystallographic texture, and dislocations density on the tensile properties were discussed.

## 2. Experiments and Methods

**Figure 1:**
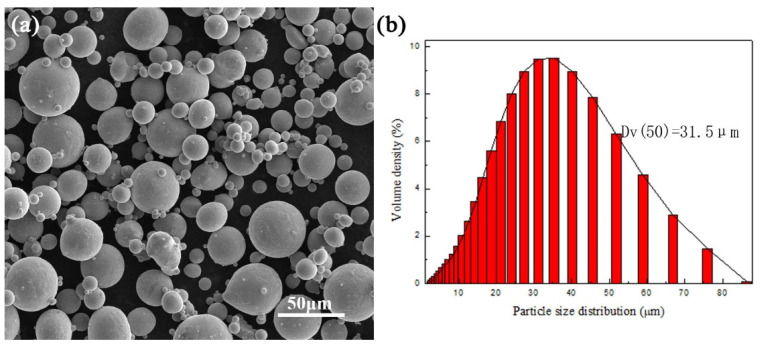
(**a**) Morphology and (**b**) the particles diameter distribution of the Ti-55511 powders.

**Figure 2:**
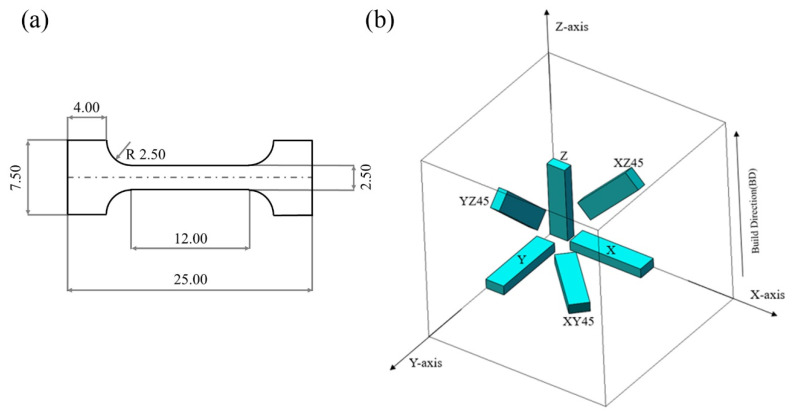
(**a**) Schematic of Ti-55511 alloy produced by SLM along with directions and (**b**) tensile specimen size (in mm).

The Ti-55511 alloy powder used in the present work was produced by gas atomization. As is shown in Table 2, the chemical composition of the powder used in this study is consistent with that of the standard Ti-55511 alloy. The morphology and the particle diameter distribution of the powder are exhibited in Figure 1a,b, respectively. A selective laser melting (SLM, Far-soon 271 M, Farsoon, Changsha, China) device equipped with a 500 W continuous-wave laser whose spot diameter ranges from 110 to 130 μm was used to manufacture the specimens with the size of 30 mm × 12 mm × 8 mm. The laser outlet was also equipped with a beam shaping mirror and an X-Y scanning galvanometer. The SLM processing parameters were: laser power = 250 W, scanning speed = 1000 mm/s, scanning distance = 120 μm, and layer thickness = 30 μm. As in previous works [25,28,32,33], a standard alternating X/Y raster scanning strategy was chosen during the fabrication by SLM. Once the layer ‘n’ was completed, the bidirectional scanning of the next layer ‘n + 1′ was performed after rotating by 67°. To avoid oxidation in the specimens, the experiments were carried out in an argon atmosphere with an oxygen concentration less than 1%. As is shown in Figure 2a, the tensile specimens were cut and then polished from the cuboids.

To explore the effects of the microstructure, crystallographic texture, and dislocation density on the mechanical behavior of the Ti-55511 alloy, two groups of specimens with the XYZ designation (X), YXZ designation (Y), ZXY designation (Z), XY45°Z designation (XY45), XZ45°Y designation (XZ45), and YZ45°X designation (YZ45) were utilized (as exhibited in Figure 2b). In particular, the first letter designates the axis parallel to the longest dimension, the second letter designates the second-longest dimension, and the third letter designates the third-longest dimension of the specimens. Based on a previous work, the optimal two-stage direct aging mechanism was applied for one group of specimens [28]. Similarly, a group of the abovementioned specimens after direct aging was marked as H-X, H-Y, H-Z, H-XY45, H-XZ45, and H-YZ45 according to the different orientations, respectively. The first step of heat treatment is holding the original sample at 750 °C for 2 h, followed by water quenching, while the second step is keeping the sample obtained in the previous step at 600 °C for 2 h followed by water quenching. Microstructure analysis was performed via an optical microscope (OM, Leica MeF3A, Leica, Wetzlar, Germany), scanning electron microscope (SEM, Quanta FEG 250, FEI, Hillsboro, OR, USA), and electron backscattered diffraction (EBSD, Helios Nanolab G3 UC Dual Beam Microscope System, FEI, Hillsboro, OR, USA). X-ray diffraction (XRD, Rigaku TTRAX III, Rigaku, Tokyo, Japan) analysis was performed with a Cu-K_α_ (λ = 1.54056 Å) radiation source operated at 40 kV and 40 mA. The results were analyzed by JADE 6. The tensile tests were carried out on a universal testing machine (TM, CMT4204, MTS, Eden Prairie, MN, USA) with a nominal strain rate of 1 × 10^−3^ s^−1^ at room temperature. Each group of tensile tests was repeated at least three times, and the average value was used to express the mechanical properties.

## 3. Results and Discussion

### 3.1. Overview

**Figure 3:**
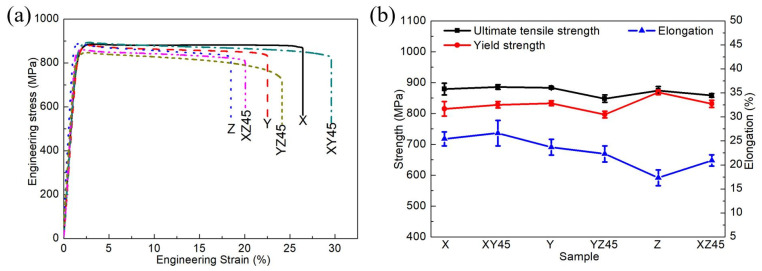
(**a**) Engineering stress–strain curves of Ti-55511 alloys. (**b**) The variations of the UTS, the YS, and the elongation at different samples.

The representative engineering stress–strain curves of the as-fabricated samples are presented in Figure 3a. The obtained samples X, Y, Z, XY45, XZ45, and YZ45 exhibit UTS values from 848 to 886 MPa, YS values from 797 to 868 MPa, Young’s modulus (E) values from 59 to 79 GPa, and elongation to failure values from 17 to 27% (Table 3). Compared with those obtained by different methods of AM (Table 1), the measured mechanical properties of the as-fabricated samples exhibit the enhanced elongation properties. As is shown in Figure 3b, the UTS, YS, and elongation of samples X, Y, and XY45 can be considered roughly the same. This indicates that the mechanical properties of the samples are almost isotropic in the XY-plane. Unlike the anisotropic values which were reported for the Young’s modulus and elongation of the SLM processed Ti-55511 by Liu et al. [22], the YS of vertical sample Z (868.2 ± 7.4 MPa) is slightly higher than that of horizontal sample X (815.0 ± 23.6 MPa); however, vertical sample Z exhibits a similar UTS (874.5 ± 13.1 MPa) and lower ductility (17.3 ± 1.6%) values compared with horizontal sample X (879.4 ± 19.2 MPa and 25.4 ± 1.5%).

**Figure 4:**
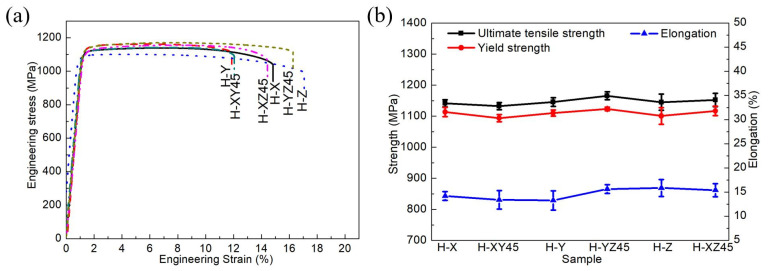
(**a**) Engineering stress–strain curves of aged Ti-55511 alloys. (**b**) The variations of the UTS, the YS, and the elongation of aged samples.

The representative engineering stress–strain curves of the aged samples are presented in Figure 4a. The samples H-X, H-Y, H-Z, H-XY45, H-XZ45, and H-YZ45 exhibit the UTS, YS, E, and elongation values from 1133 to 1166 MPa, 1093 to 1123 MPa, 98 to 111 GPa, and 13 to 16%, respectively (Table 4). Comparing these results with the SLMed samples’ results (as shown in Table 3), the mechanical properties of the aged samples demonstrate a higher tensile strength and elasticity modulus, but lower elongation than those of the SLMed samples. In addition, the mechanical properties of the aged samples in all orientations meet the aerospace allowable properties (YS of 1010 MPa and elongation of 8%) [19,23]. As is shown in Figure 4b, the UTS, YS, and elongation values of the aged samples can be considered roughly the same. Regardless of the anisotropic properties of the AMed Ti-55511 alloy (as depicted in Table 1), the mechanical properties of the aged Ti-55511 samples are almost isotropic. In order to deeply discuss the anisotropic properties, microstructural characterizations will be presented in the following section.

### 3.2. Phase Analysis

#### 3.2.1. As-Fabricated Samples

**Figure 5:**
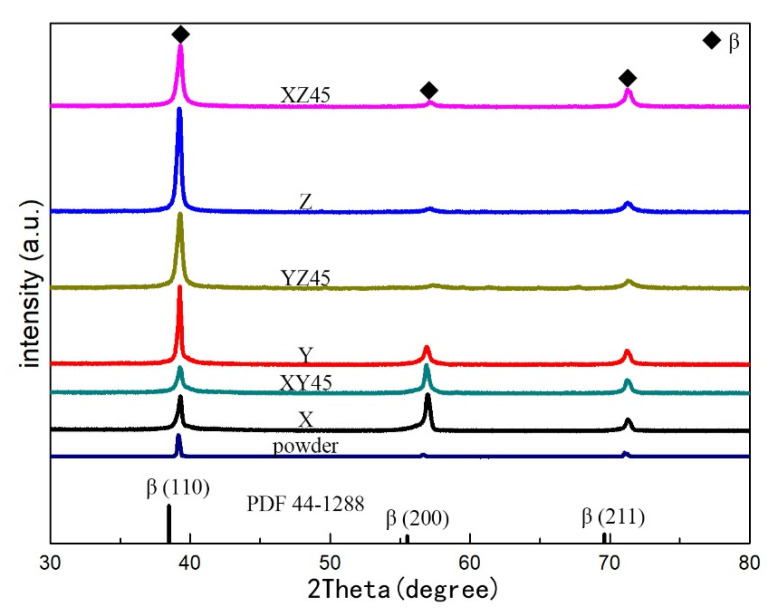
The XRD spectra of pre-alloy powder and as-fabricated samples of Ti-55511.

The X-ray diffraction patterns of the pre-alloy powder and as-fabricated samples are shown in Figure 5. After the SLM process, only the β phase was detected, which is the same as in a previous work [25]. The β diffraction peaks shift to higher angle in all the samples compared with the diffraction peaks of the β phase in the standard PDF cards (No. 44–1288).

The standard β phase presents a cubic structure with the lattice parameter of *a* = 3.3065 Å according to the standard PDF cards (No. 44–1288). For a peak measured at 2θ using monochromatic X-rays, the corresponding plane spacing can be determined using Bragg’s law [34]:

*nλ* = 2*dsinθ* (1)

where *λ* is the wavelength of the monochromatic X-ray (1.54056 Å in the present study), *θ* is the Bragg angle, and *d* is the plane spacing of the {*hkl*} plane.

For the cubic crystal, the interplanar distance *d* can be given as follows [32]:

(2) $$d_{\mathrm{hkl}}=\frac{a}{\sqrt{h^{2}+k^{2}+l^{2}}}$$

where *a* is the lattice constant and *h*, *k*, and *l* are Miller indices.

For *n* = 1, Equation (3) can be written as below from Equations (1) and (2):

(3) $$a=\frac{\lambda }{2sin\theta_{hkl}}\sqrt{h^{2}+k^{2}+l^{2}}$$

The below Scherrer relation was used for the calculation of the crystallite size of samples [35,36]:

(4) $$D=\frac{K\lambda }{FWHM\cdot cos\theta }$$

where *λ* is the wavelength of the monochromatic X-ray, *D* is the crystallite size, *θ* is the Bragg angle, *FWHM* (abbreviated from full width at half maximum) is the broadening at half of the maximum intensity, and *K* is the shape factor (0.89 in the present study) [36,37]. In particular, the peak belonging to the *FWHM* of the XRD pattern comes from two parts: the first part is the peak width of the instruments which can be detected and eliminated by fully annealed Si powder, and the other part is the peak width caused by the crystal structure characteristics such as the grain size and the dislocation density of the material. According to the Scherrer equation given above, the crystallite size is inversely proportional to the *FWHM* value.

The micro-strain (*ε*) [36,38], dislocation density (*δ*) [39], and intensity ratio (*f*) [40] of the as-fabricated samples were also estimated from the following relations and are given in Table 5:

(5) $$\varepsilon =\frac{FWHM}{4tan\theta }$$

(6) $$\delta =\frac{2\sqrt{3}\varepsilon }{Db}=\frac{\sqrt{3}}{2b}\frac{FWHM^{2}cos\theta }{K\lambda tan\theta }\;$$

(7) $$f_{hkl}=\frac{I_{hkl}}{\sum I_{hkl}}\;$$

where *b* is the magnitude of the Burgers vector (about 0.280 nm for β crystallite [41] and 0.295 nm for α crystallite [42]), *D* is the crystallite size, and *I* is the intensity of the XRD peak. For the β phase, the $\sum I_{hkl}$ of Equation (7) equals to the sum of *I*_(110)_, *I*_(200)_, and *I*_(211)_.

The value *f*_(110)_ of the strongest XRD peak is 77.519% due to the standard PDF cards (No. 44–1288). To reflect the crystallite parameters more accurately, *D*, *ε*, and *δ* were calculated as the average value of all the diffraction peaks, which is comparable with the β-Ti alloys (*ε* = ~4.886 × 10^−3^ and *δ* = ~2.014 × 10^15^ m^−2^) [41]. As is shown in Table 5, the calculated parameter *a* is 3.24 to 3.25 Å for the as-fabricated samples. According to Equation (3), *θ* increases as *a* decreases. As is shown in Figure 5 the reason for the slight shifts in the Bragg angle should be the disordering created by positioning the atoms with different diameters in the lattice. The atomic radius of Al, Mo, V, Cr, Fe, and Ti are 1.43, 1.39, 1.34, 1.28, 1.26, and 1.47 Å [43], respectively. Since the β solid-solution phase contains a large number of elements with small atomic radii, the lattice constant of β in the SLMed Ti-55511 alloy is smaller than that of pure β titanium.

In addition, the micro-strain of crystallite may also cause slight shift in the Bragg angle. The average crystallite sizes of as-fabricated samples were calculated as 18 to 21 nm (Table 5). As is shown in this table, the micro-strain and dislocation density after the SLM process are increased significantly due to the decrease in crystal size as well as the increase in the *FWHM* value of the XRD peak intensity. This indicates that a large number of dislocations are created within the samples after rapid cooling of SLM process, leading to the generation of microscopic strain.

In order to quantitatively evaluate the degree of alignment in the building direction [40], it was calculated that the intensity ratio *f*_(110)_ of the powder and samples at the X, Y, Z, XY45, XZ45, and YZ45 directions are 76.047%, 38.522%, 71.272%, 89.574%, 33.711%, 71.185%, and 88.354%, respectively. When the value *f*_(110)_ is greater than 77.519%, it implies that the fiber texture formed in the SLM process [9,24,44]. The texture of different as-fabricated samples varies greatly.

#### 3.2.2. Aged Samples

**Figure 6:**
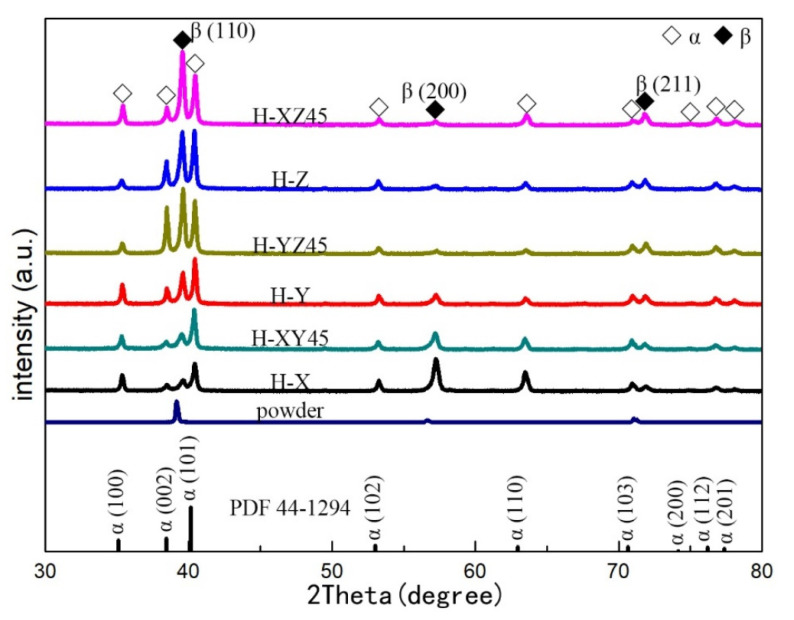
The XRD spectra of pre-alloy powder and aged samples of Ti-55511.

After direct aging treatment, the diffraction peaks of the α and β phases without new phase separation can be observed in the spectrum of the SLMed Ti-55511 as shown in Figure 6. The XRD spectra of the aged samples are similar to that of a hot rolled near-β Ti-55511 alloy [45]. The β diffraction peak shifts to a higher angle in samples compared with that of the powder, which indicates that the lattice spacing of β decreases due to the segregation of Mo, V, Cr, and Fe in the β phase [25].

Moreover, the structural parameters of the prior-β phase were calculated and are shown in Table 6. The micro-strain and dislocation density created in the prior-β phase were decreased significantly after direct aging. Meanwhile, the micro-strain and dislocation density in all of the as-fabricated samples are very similar.

The standard α phase presents a hexagonal structure with the lattice parameters of *a* = 2.9505, *c* = 4.6826 Å, and *c*/*a* = 1.587 according to the standard PDF cards (No. 44–1294). The interplanar distance *d* for a hexagonal crystal structure can be given by the following relation [33]:

(8) $$\frac{1}{d_{hkl}^{2}}=\frac{4}{3}\left(\frac{h^{2}+hk+k^{2}}{a^{2}}\right)+\frac{l^{2}}{c^{2}}$$

where *a* and *c* are the lattice parameters and *h*, *k*, and *l* are Miller indices. For *n* = 1, Equations (1) and (8) can be written as

(9) $$sin^{2}\theta =\lambda^{2}\left(\frac{h^{2}+hk+k^{2}}{3a^{2}}+\frac{l^{2}}{4c^{2}}\right)$$

For the (002) plane, where *h* = 0, *k* = 0 and *l* = 2, Equation (9) can be written as

(10) $$c=\frac{\lambda }{sin\theta_{002}}$$

For the (101) plane with the strongest XRD peak, Equation (9) can be written as

(11) $$a=\frac{2\lambda c}{\sqrt{12c^{2}sin^{2}\theta_{101}-3\lambda^{2}}}$$

For the α phase, the $\sum I_{hkl}$ of Equation (7) equals to the sum of *I*_(100)_, *I*_(002)_, *I*_(101)_, *I*_(102)_, *I*_(110)_, *I*_(103)_, *I*_(200)_, *I*_(112)_, and *I*_(201)_, which indicates the sum of intensities belongs to XRD peaks from 30 to 80 degrees. The value *f*_(101)_ belonging to the strongest XRD peak is 48.544% according to the standard PDF cards (No. 44–1294). According to Equations (1)–(11), the structural parameters of the α phase are presented in Table 7. Martensitic α′ is conveniently observed as a distorted α-hcp structure with small lattice parameters (*a* = 2.931 Å, *c* = 4.681 Å, and *c*/*a* = 1.597) [46]. The calculated *c*/*a* of the α phase is roughly 1.597 for the aged samples, which implies that the martensitic α′ was formed after direct aging. The micro-strain and dislocation density values of the α phase are close to those of the β-matrix. Meanwhile, the micro-strain and dislocation density of all aged samples are very similar owning to the fact that the α phase was transformed from the prior β phase following the Burgers orientation relationship (BOR), that is, {001}_α_//{110}_β_ and <110>_α_//<111>_β_, and a near-random distribution of 12 variants of the α phase was also formed [47,48]. After direct aging, it can be seen that the *I*_(110)_ value of the prior β phase is generally reduced by comparing the intensity ratio of *f*_(110)_ in Table 5 and Table 6. However, the *f*_(101)_ value of the strongest XRD peak is slightly below that of the standard α phase in Table 7, which implies a weak texture in the transformed microstructure. It is reported that only the weak texture of the α phase was detected due to all of the 12 possible α variants being randomly distributed with the Burgers orientation relationship between the prior-β and transformed α phase in AMed materials [5,47,48,49]. A more detailed investigation for the texture was conducted by EBSD, which will be discussed in the following part.

### 3.3. Microstructural Characteristics

#### 3.3.1. As-Fabricated Samples

**Figure 7:**
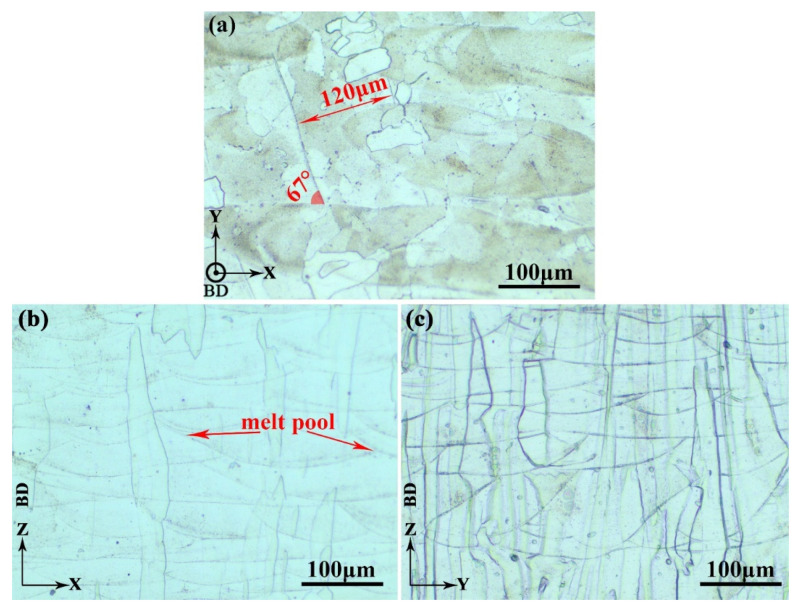
The OM images showing typical microstructures of sample X in the (**a**) XY-, (**b**) XZ-, and (**c**) YZ-planes. BD represents the build direction.

The XY-, XZ- and YZ-planes of sample X were used to characterize the microstructure of the as-fabricated Ti-55511 alloy in this study. Figure 7a–c show the optical microscopy (OM) images of the Ti-55511 alloy in the XY-, XZ-, and YZ-planes, respectively. The laser tracks are visible in the OM images. As depicted in Figure 7a, a regular laser melted tracks with the alternating 67° filling strategy, which is observed on the cross-section. During the SLM process, the temperature at the center of the molten pool would be more than 3000 °C [50]. The laser scanning speed is 1000 mm/s and the cooling rate reaches 10^3^ to 10^8^ k/s [51]. Consequently, the Ti-55511 powder experiences a complete process of melting and then nonequilibrium solidification. The average width (120 μm) of the laser scanning tracks equals to the hatch distance (120 μm), as is shown in Figure 7a. As demonstrated in Figure 7b,c, all the β columnar grains are parallel to the build direction, which is consistent with previous works [24,25]. According to the columnar grain evolution during SLM [25], the melt pools will be retained in the as-fabricated samples. Moreover, the melt pools are approximately 150 μm in width in Figure 7b, which is larger than the width of laser scanning of each track. A part of the material in each track would be remelted when the laser moves up to an adjacent track, which represents that the microstructure displays partial remelting between adjacent tracks. As the beam moves along its build path, the melt pool is created along remelting both in the current surface layer as well as the previously deposited layer [52]. The remelting process affects the texture, which was detected in a previous work [25].

**Figure 8:**
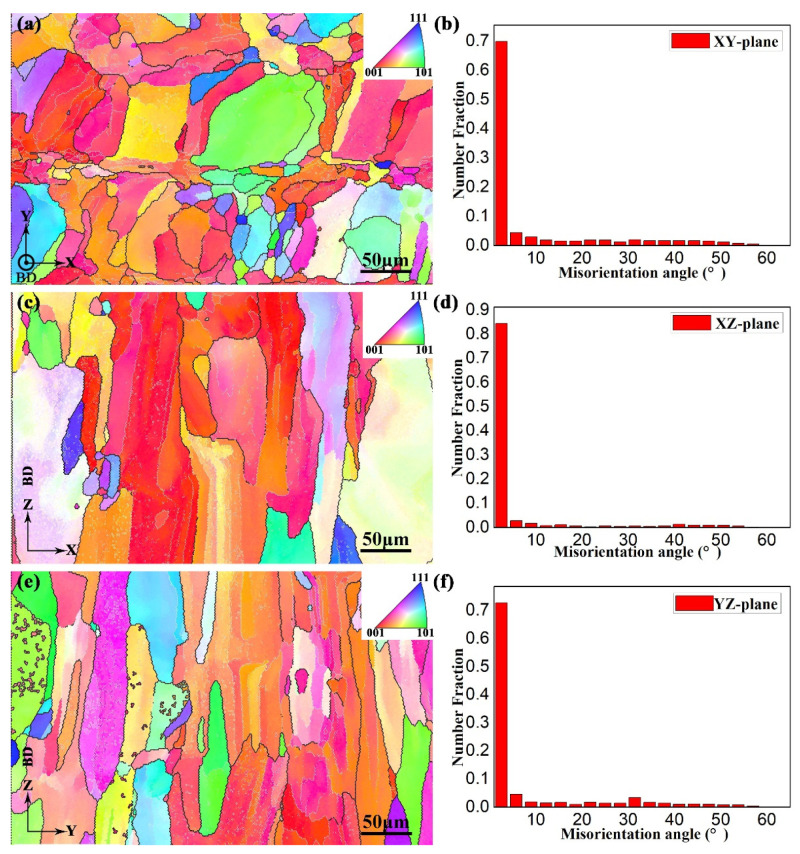
The EBSD inverse pole figure (IPF) maps of sample X at (**a**) XY-, (**c**) XZ-, and (**e**) YZ-planes, respectively. Misorientation angle distribution at (**b**) XY-, (**d**) XZ-, and (**f**) YZ-planes, respectively.

The EBSD maps of the as-fabricated samples at the XY-, XZ-, and YZ-planes are demonstrated in Figure 8a,c,e, respectively. The laser scanned tracks are occupied by fine grains as shown in the EBSD maps, which is consistent with the OM images (Figure 7). For the XZ- and YZ-planes, Figure 8c,e exhibit that the microstructures consist of mostly the β columnar grains which grow across the multiple melt pools along the building direction. High-angle grain boundaries (HAGBs) are depicted with black (Figure 8a,c,e). As is shown in Figure 8a, the finer grains are distributed in melt tracks, and the coarser grains are distributed between the adjacent melt tracks. Figure 8b,d,f reflect the boundary misorientation distribution of the XY-, XZ-, and YZ-planes, respectively. The distribution of misorientation angles of the SLM-fabricated Ti-55511 alloy is selectively oriented (most of the misorientation angles are less than 10°), which indicates that the SLM-fabricated Ti-55511 alloy possess anisotropic properties [7,22].

**Figure 9:**
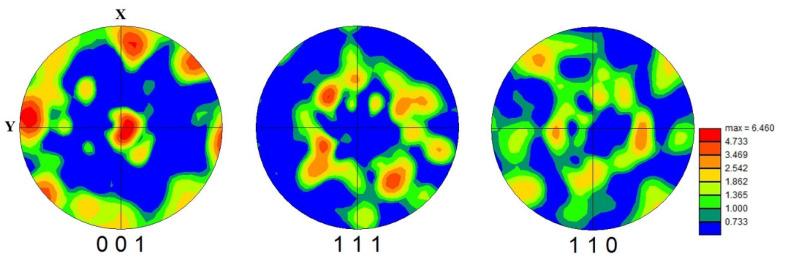
The EBSD pole figure (PF) maps of the β phase in sample X.

In order to verify the crystallographic orientation of β grains, further investigations with the EBSD are shown in Figure 9. According to the mechanism of texture evolution during the SLM process [40], the β textures display mainly a simple {001} fiber texture with a maximum intensity of about 6.46 times that of the random one. The rapid solidification across the interface between the boundaries of the primary-solidified area and resolidified area were discussed in detail in a previous work [25], which may lead to a lower degree of texture formation.

#### 3.3.2. Aged Samples

**Figure 10:**
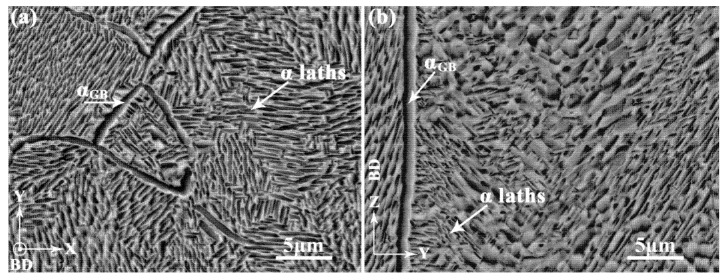
The SEM images of sample H-X in (**a**) XY- and (**b**) YZ-planes.

In order to perform comparative analysis, sample H-X was used to characterize the microstructure of the aged Ti-55511 alloy in this study. For metastable β titanium alloys, post-build aging has been used to give them high strength and moderate ductility via controlling the morphology, dimension, and volume fraction of the α phase [34,53]. This has been confirmed by a previous work [28]. Therefore, much attention should be paid to the influence of the α phase on relative mechanical properties. Since the XZ- and YZ-planes depicted similar microstructures in the previous Section 3.3.1, microstructures of the XY- and YZ-planes are utilized for analysis as shown in Figure 10a,b, respectively. According to the XRD analysis (Figure 6), a large number of α laths exist in the XY- and YZ-planes of the aged samples. At the same time, the α_GB_ also exists at the prior-β grain boundaries according to the previous work [28]. The α laths seem to have no fixed crystal orientation either in the XY- or YZ-planes, which indicates a weak texture in the microstructure, as shown in Figure 10a,b. In particular, no significant β columnar grain is observed, indicating a large number of β phase are transformed to α laths in the aged samples. A more detailed investigation for the phase was conducted by EBSD, which will be discussed in the following part.

**Figure 11:**
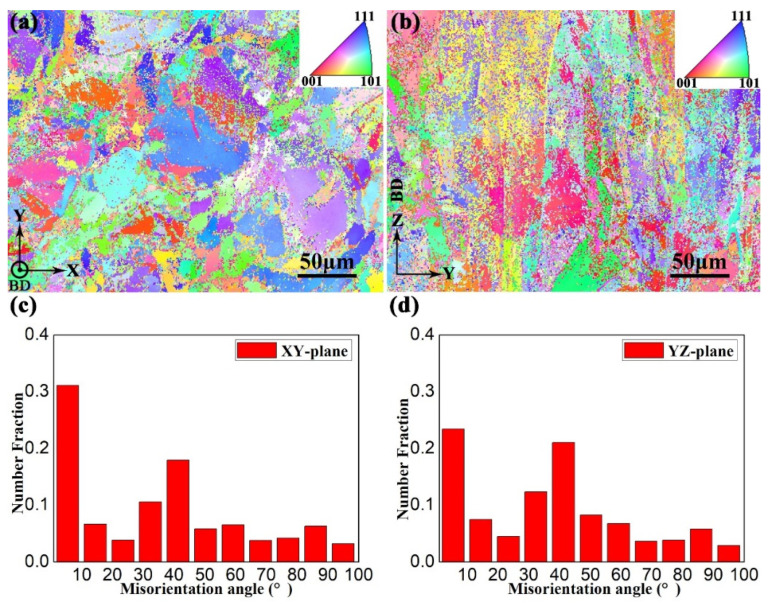
The EBSD inverse pole figure (IPF) maps of sample H-X at (**a**) XY- and (**b**) YZ-planes. Misorientation angle distribution at (**c**) XY- and (**d**) YZ-planes.

The EBSD maps of the aged samples at the XY- and YZ-planes are shown in Figure 11a,b, respectively. Although a large number of β phases were transformed to α laths, the prior-β grains still remained in the XY- and YZ-planes. This is depicted in Figure 11a,b. Combined with the XRD results (Figure 6), the α phase content of sample H-X, which can be obtained by EBSD analysis, is about 76.9%. Figure 11c,d are the boundary misorientation distribution of the XY- and YZ-planes, respectively. After the aging treatment, the fraction of LAGBs (lower than 15°) were reduced from 79.2% to 31.1% in the XY-plane and 80.7% to 30.9% in the YZ-plane, respectively. This implies the selective orientation of misorientation angles was greatly reduced. According to the XRD analysis, the prior-β phase texture is weakened after the aging treatment due to the nucleation of the α phase with weak texture [10,11]. This could be a reason for the reduction of LAGBs and selective orientations.

**Figure 12:**
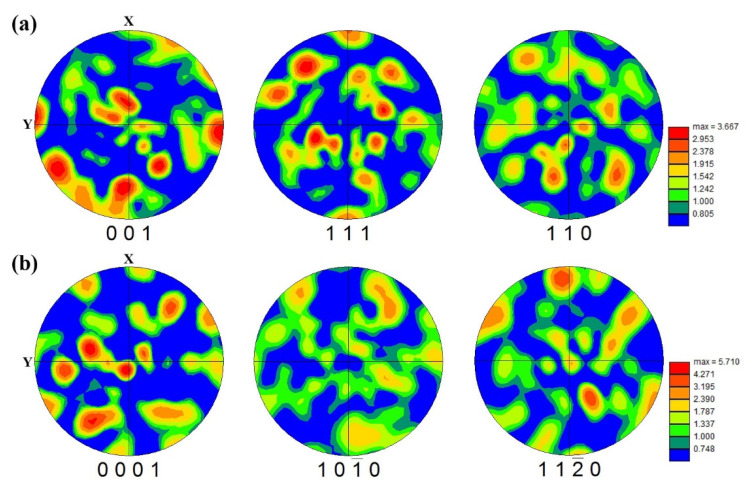
The EBSD pole figure (PF) maps of sample H-X for (**a**) β and (**b**) α phases.

To confirm the weak texture, further investigations performed with EBSD and the corresponding results are represented in the pole figures of Figure 12. The maximum intensity of texture for the β phase was reduced from 6.460 times (Figure 9) to 3.667 times (Figure 12a) that of the random one. The reduction of texture at the β phase is consistent with the XRD analysis, which is shown in Figure 6. Meanwhile, Figure 12b illustrates the weak texture of α laths with the maximum intensity of 5.710 times that of the random one.

**Figure 13:**
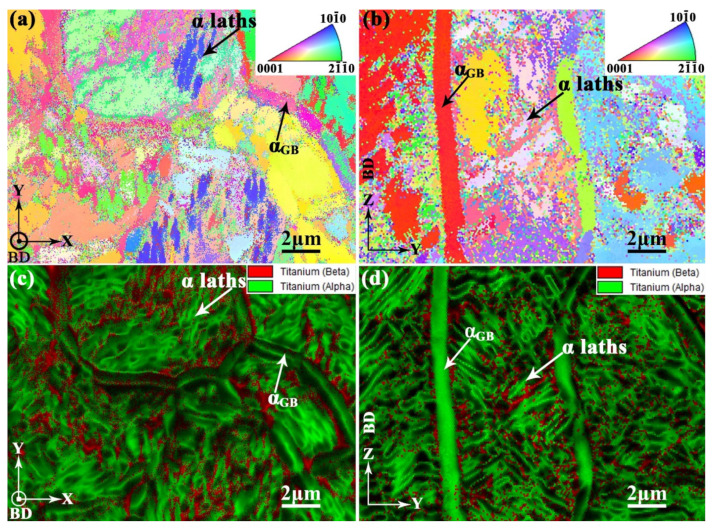
High magnification of EBSD pole figure (PF) maps of sample H-X at (**a**) XY- and (**b**) YZ-planes, respectively; the diffraction pattern qualities and phase maps at (**c**) XY- and (**d**) YZ-planes, respectively.

As is shown in Figure 13a,b, various crystallographic orientations of α laths can be clearly seen at the XY- and YZ-planes of sample H-X, which indicates the weak texture of α laths. The weak texture of α laths may be due to the near-random distribution of 12 α phase variants from BOR transformation [5,47,48,49]. Moreover, the α_GB_ was found at the XY- (Figure 13c) and YZ-planes (Figure 13d) of sample H-X. The crystallographic orientations of α_GB_ are always inconsistent with those of the surrounding α laths. The effects of microstructure and crystallographic texture on the tensile properties are discussed in the following sections.

### 3.4. Tensile Properties

#### 3.4.1. As-Fabricated Samples

The YS of vertical sample Z is slightly higher than that of horizontal sample X in the current study; however, vertical sample Z exhibits a similar UTS and lower ductility compared with horizontal sample X. The slight anisotropy is due to the formation of β columnar grains with a body-centered cubic (BCC) crystal structure. Concerning the Schmid factor, the yield strength of a material at the macro-level can be given as follows [32]:

(12) $$\sigma_{\mathrm{YS}}\;\;\geq \;\frac{\;\tau }{cos\varphi \;cos\;\lambda }$$

where *φ* is the angle between the axial tensile force and the normal direction of the slip plane, *λ* is the angle between the axial tensile force and slip direction, cos*φ* cos*λ* is the Schmid factor (*μ*), and *τ* is the critical resolved shear stress in the slip systems, which only depends on the nature of the material. In BCC materials, the slip system is {110}〈111〉, and the sum of *φ* and *λ* is 90°. Therefore, Equation (12) can be written as follows:

(13) $$\sigma_{\mathrm{YS}}\;\geq \;\frac{\;\tau }{\frac{1}{2}sin2\lambda }$$

The Schmid factor *μ* can be calculated as $\frac{1}{2}sin2\lambda$. A large value of *μ* indicates a low yield strength of a material. As shown in Figure 7 and Figure 8, the β columnar structure with {001} fiber texture was observed in the Ti-55511 samples processed by SLM. This illustrates that the angle between the slip system and axial tensile force is 54.7° in both the horizontal and vertical samples, while it is 35.3° in the angled (45°) samples. Accordingly, the Schmid factor *μ* can be calculated as follows [9]:

(14) $$\mu =\frac{1}{2}sin\left(2\times 54.7{}^{\circ}\right)\approx 0.47$$

(15) $$\mu =\frac{1}{2}sin\left(2\times 35.3{}^{\circ}\right)\approx 0.47$$

The Schmid factor value *μ* for the horizontal, vertical, and angled (45°) samples is the same, which may lead to the same yield strength. The theoretical model cannot explain the experimental results of this study, which indicates that the β crystal structure may not be the main cause of the slight anisotropy.

The anisotropy of AMed Ti-55511 alloy was discussed in detail by Wang et al. [7,22]. The angles between the ensile load direction and the columnar grain boundaries direction determined the cracking mechanism. The grain boundary density was encountered by dislocation motion at various angles. The tensile strength was increased by the dislocation density. The anisotropy of the ductility can be attributed to the different cracking mechanisms between horizontal and vertical samples subjected to tension stress. Thus, dislocation strengthening is proposed to explain the experimental results of the current study. Considering the anisotropy of dislocations distribution due to the texture, the Taylor hardening is used for evaluating the dislocation strengthening as follows [42,54]:

(16) $$\sigma_{\mathrm{YS}}=\sigma_{0}+MaGb\sqrt{\delta }$$

where $\sigma_{0}$ is the lattice strength [42], *M* is the Taylor factor (α-Ti: 0.85, β-Ti: 2.8) [41,55], *α* is the geometrical factor (α-Ti: 0.2, β-Ti: 0.3) [41,55], *G* is the shearing modulus (α-Ti: 45.6 GPa, β-Ti: 36 GPa) [55], *b* is the Burgers vectors (α-Ti: 0.295 nm, β-Ti: 0.280 nm) [41,55], and *δ* is the total dislocation density of geometrically necessary dislocations (GNDs) and statistically stored dislocations (SSDs) [54], which can be estimated via XRD [56]. According to Equation (16), $\sigma_{YS}$ will increase with the increasing of the dislocation density. The different contributions of dislocation strengthening between the vertical Z and horizontal X samples can be estimated at about 80.4 MPa due to Equation (16) and Table 5. The value of *α* decreases with increasing of the dislocation density [57]. The ultimate tensile strength can be given as follows:

(17) $$\sigma_{\mathrm{UTS}}=\sigma_{\mathrm{YS}}\;+\;\sigma_{\mathrm{Plastic}}$$

where $\sigma_{\mathrm{YS}}$ and $\sigma_{\mathrm{Plastic}}$ are the yield strength (elastic strength) and plastic strength, respectively. Many studies have been carried out on the plastic deformation of metals, finding that the mechanical plastic responses are highly affected by the development, multiplication, accumulation, and migration of dislocations [58,59,60]. GNDs accumulate in plastic strain gradient fields to maintain the strain compatibility across microstructures, which can be calculated as follows [30,61]:

(18) $$\sigma_{\mathrm{Plastic}\;}=MCaGb\sqrt{\delta_{\mathrm{GND}}}$$

where $\delta_{\mathrm{GND}}$ is the density of GNDs, *M* is the Taylor factor, *C* is a constraint factor, *a* is an empirical constant [62,63], *G* is the shearing modulus, and *b* is the magnitude of the Burgers vector. Similar to Equation (16), the Taylor factor *M*, constraint factor *C*, empirical constant *a*, shearing modulus *G,* and Burgers vector *b* should be different before and after the aging treatment.

**Figure 14:**
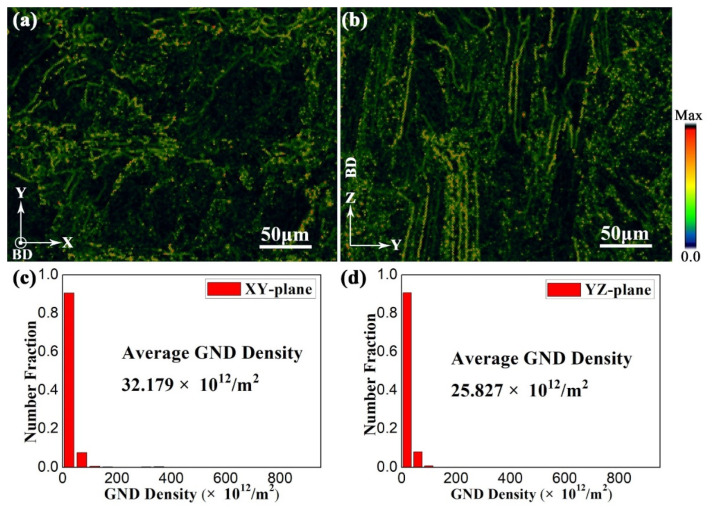
The EBSD maps of geometrically necessary dislocations (GNDs) with the corresponding distribution in sample X at (**a**,**c**) XY-, and (**b**,**d**) YZ-planes.

The GNDs and the corresponding distribution in sample X at the XY- (Figure 8a) and YZ-planes (Figure 8c) are displayed in Figure 14. The GND density can also be estimated according to the results of Dai et al. [64]. The average GND density of sample X in the XY- and YZ-planes is 32.18 × 10^12^ m^−2^ and 25.83 × 10^12^ m^−2^, respectively. Moreover, the number fraction of the GNDs is highly concentrated at small dislocation densities. According to Equation (18), the plastic strength is increased with the GND density. This could be considered as a reason why the plastic strength of horizontal sample X is greater than that of vertical sample Z (Figure 3b).

**Figure 15:**
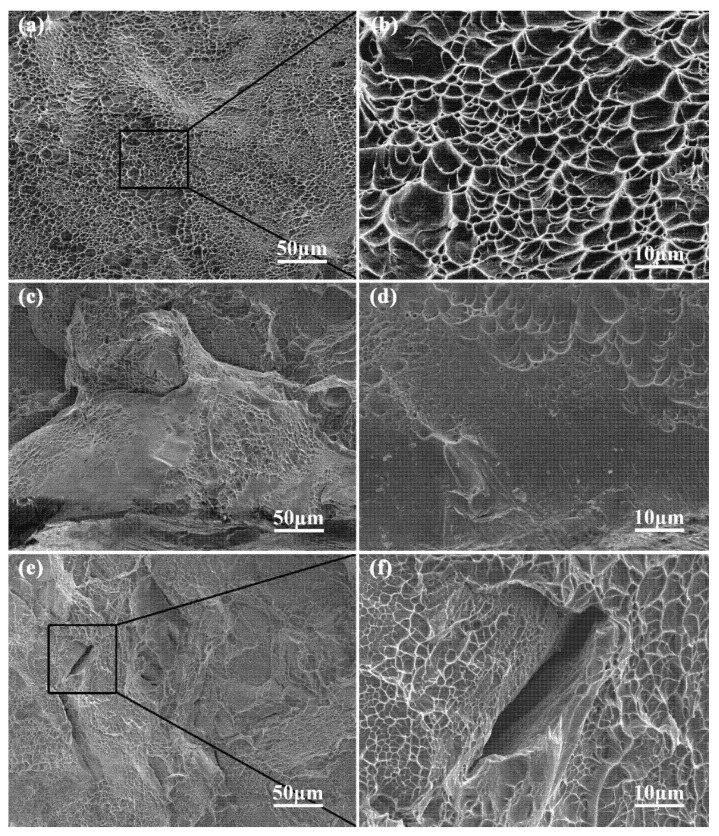
The SEM images of fracture surfaces of SLM processed Ti-55511: (**a**,**b**) horizontal sample X and (**c**–**f**) vertical sample Z at room temperature.

As is shown in Figure 15, the fracture surfaces of horizontal sample X and vertical sample Z mainly exhibit dimpled surfaces. Figure 15b,d,f depict the magnified images of the fracture surfaces. Horizontal sample X displays mainly intergranular fracture, while the intergranular fracture as well as transgranular fracture appear in vertical sample Z. When the columnar β grains are oriented parallel to the loading direction (i.e., vertical sample), any crack following the β/β grain boundaries would have to follow a tortuous way to pass through large β/β grain boundaries. Therefore, these do not facilitate an easy path for the crack propagation due to the increasing of the grain boundaries volume fraction. Then, ductile transgranular crack propagation occurs instead of the coalescence of microvoids nucleated within the intragranular slip bands. This can be confirmed by the uneven fracture surfaces and also the ductile fracture voids in Figure 15c. At the same time, the fracture surfaces without a ductile mode of fracture can also be observed in Figure 15c,d, which may originate from the layer-by-layer nature of the SLM process as well as the tensile residual stress. Whereas the tensile residual stress is converted into compressive stress and the compressive stress is enhanced by multiple thermal cycles [65,66,67], the bonding ability is decreased between neighboring layers and thus decreases the ductility. Hence, vertical sample Z (consisting of the highest number of layers) has the minimum ductility. Moreover, the melted alloy in one layer does not completely fill the gaps between different passes in previous layers. The sharp angles in lack-of-fusion (LOF) pores (Figure 15e) results in the local stress concentration during loading. The pores have been proved to play a significant role in early fracture, particularly in the vertical samples [68]. Vertical sample Z is the most critical sample because the loading direction opens up the defect (Figure 15f). The lower GND density, tensile residual stress, and layer-by-layer bonding strength could be considered as the reasons for the lower ductility of the as-fabricated vertical sample Z [58]. Moreover, the tensile residual stress caused by layer-by-layer deposition may also be a reason for the finding that the micro-strain and dislocation density of vertical sample Z is larger than that of horizontal sample X (as illustrated in Table 5).

#### 3.4.2. Aged Samples

The ultimate and yield strengths of the aged samples are 1133~1166 MPa and 1093~1123 MPa, respectively. These are significantly higher than those of the as-fabricated samples, which are 848~886 MPa and 797~868 MPa, respectively. However, the aged samples exhibit a lower ductility (13~16%) compared with the as-fabricated samples (17~27%). The mechanical properties of the aged Ti-55511 samples are almost isotropic. The differences are likely due to the α and β-matrix structure with a weak texture (Figure 12), resulting in the anisotropy property being weaker than that of the single β columnar structure. Similarly, considering the dislocation glide is impeded by two sets of obstacles, the following superposition law is obtained [24]:

(19) $$\delta \;=F_{V}^{\alpha }\delta_{\alpha }+F_{V}^{\beta }\delta_{\beta }$$

where $F_{V}^{\alpha }$ and $F_{V}^{\beta }$ are the volume fraction of the α and β phases and $\delta_{\alpha }$ and $\delta_{\beta }$ are the total dislocation density of the α and β phases, respectively. Based on the total dislocation density of the α and β phases (Table 6 and Table 7) and the volume fraction of the β (~23.1%) and α phases (~76.9%), the different contributions of dislocation strengthening between the vertical H-Z and horizontal H-X samples can be estimated at about 8.2 MPa. This is consistent with the present work (Table 4). Furthermore, when the models of Wang et al. [4,7,22] are used to explain the mechanical anisotropy properties of AM alloys, it should be noted that there may be some deviations or even errors (i.e., the tensile strength of the angled (45°) sample in the current work).

**Figure 16:**
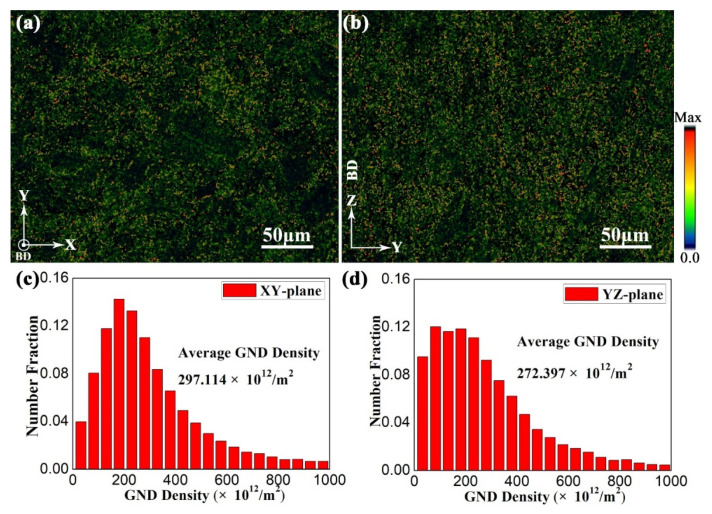
The EBSD maps of the GNDs with the corresponding distributions in sample H-X at (**a**,**c**) XY-, and (**b**,**d**) YZ-planes.

The GNDs and the corresponding distribution in sample H-X at the XY- and YZ-planes are displayed in Figure 16, which is extracted from the EBSD analysis of sample H-X (Figure 11). The average GND density of sample H-X at the XY- and YZ-planes is 297.114 × 10^12^ m^−2^ and 272.397 × 10^12^ m^−2^, respectively. Moreover, the number fraction of the GNDs is more uniform than that of the as-fabricated sample. It should be noted that the total dislocation density consists of GND density and SSD density. The GND density increases with the precipitation of α laths after direct aging treatment (Figure 14 and Figure 16). Due to the extremely high cooling rate of the SLM process, the multilayer alloy is rapidly solidified in the melt pool to form a nonequilibrium microstructure with a large lattice distortion energy (Table 5). After the aging treatment, the strain relaxation is mainly achieved by the formation of GNDs during the transition from β to α laths, which has been confirmed by the decrease in micro-strain (Table 5 and Table 6) and increase in GND density (Figure 14 and Figure 16).

It is assumed that the anisotropy degree of tensile strength can be estimated by Equation (20) as follows:

(20) $$g_{strength}\;=(\sqrt{\delta_{max}}-\sqrt{\delta_{min}})/\sqrt{\delta_{max}}$$

where $\delta_{max}$ and $\delta_{min}$ are the maximum and minimum value of the total dislocation density in the samples with different orientations. According to Equation (20), the anisotropy degree $g_{strength}$ of the samples before and after the aging treatment can be calculated as 0.170 and 0.050, respectively. This indicates that the tensile strength anisotropy of samples is greatly weakened after the aging treatment. This is consistent with the change of texture which is demonstrated in Figure 12. According to Equations (16), (19), and (20), the different contributions of dislocation strengthening ($\sigma_{\mathrm{YS}}$) between vertical H-Z and horizontal H-X samples can be ignored. This is consistent with the change in yield strength which is illustrated in Figure 4.

**Figure 17:**
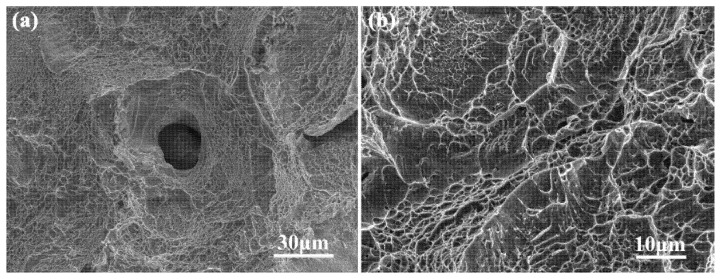
The (**a**) low and (**b**) high magnification SEM images of fracture surfaces of vertical sample H-Z of SLMed Ti-55511 alloy at room temperature.

All the fracture surfaces of aged samples exhibit dimpled surfaces, cleavage surfaces, and tortuous crack growth. The SEM images of fracture surfaces of vertical sample H-Z is shown in Figure 17. The crack was initiated and grew along the precipitate-free zones (PFZ) of the α_GB_ and α phases, which is discussed in detail in a previous work [28]. The crack propagation decreases the fracture ductility of aged samples compared with as-fabricated samples. The intergranular and transgranular fractures appear in vertical sample H-Z (Figure 17a,b); however, those without ductile fracture cannot be observed. This indicates that the decreasing of tensile residual stress increases the GND density and also the layer-by-layer bonding strength due to the precipitation of α laths after the aging. Consequently, the precipitation increases the fracture ductility of vertical sample H-Z. Compared with the as-fabricated sample Z, the plastic strength of vertical sample H-Z increases after the aging treatment. This can be confirmed by the plastic strength of as-fabricated vertical sample Z as well as aged vertical sample H-Z (Figure 3 and Figure 4).

## 4. Conclusions

The anisotropic microstructure and mechanical properties of the SLMed Ti-55511 alloy before and after the aging treatment have been studied. The primary conclusions are as follows:

1. The SLMed Ti-55511 alloy before the aging treatment exhibits slight anisotropy in its mechanical properties. The orientations within the XY-plane have a shallow effect on the mechanical properties. The yield strength of the vertical samples (868.2 ± 7.4 MPa) is slightly higher than that of the horizontal samples (815.0 ± 23.6 MPa); however, the vertical samples exhibit a similar ultimate tensile strength (874.5 ± 13.1 MPa) and lower ductility (17.3 ± 1.6%) compared with the horizontal samples (879.4 ± 19.2 MPa and 25.4 ± 1.5%).
2. The anisotropy of the SLMed Ti-55511 alloy reduces after the aging treatment. The ultimate tensile strength and yield strength of the aged samples (1133~1166 MPa and 1093~1123 MPa) are significantly higher than those of as-fabricated samples (848~886 MPa and 797~868 MPa); however, the aged samples exhibit a lower ductility (13~16%) compared with the as-fabricated samples (17~27%). In the meantime, the mechanical properties of the aged samples meet the aerospace allowable specification well.
3. The as-fabricated Ti-55511 alloy exhibits a single β columnar structure with {001} fiber texture. The vertical samples (3.1 × 10^15^ m^−2^) exhibit a higher total dislocation density compared with the horizontal samples (2.2 × 10^15^ m^−2^), which results in an enhancement of the yield strength. In addition, the weak ductility of the vertical samples may be due to the low bonding ability between neighboring layers.
4. The aged Ti-55511 alloy exhibits α and prior-β structures with a weak texture. The total dislocation density of the aged samples (~134.8 × 10^13^ m^−2^) is significantly lower than that of the as-fabricated samples (~259.4 × 10^13^ m^−2^); however, the aged samples exhibit higher GND density (~28.5 × 10^13^ m^−2^) compared with the as-fabricated samples (~2.9 × 10^13^ m^−2^). Thus, a new approach to strengthening theory is proposed to explain the anisotropic mechanical properties of AM alloys, which was confirmed by the experimental investigations on the SLMed Ti-55511 alloy before and after the aging treatment.

## Figures and Tables

**Table 1:** A summary of mechanical properties of relevant Ti-5Al-5Mo-5V-1Cr-1Fe alloys reported in the literature.

**Type of AM Process**	**Condition**	**Specimen Orientation**	**Yield** **Strength (MPa)**	**Ultimate Tensile** **Strength (MPa)**	**Elongation (%)**	**Reference**
LMD	As-built	Vertical	-	1178 ± 20	5 ± 0.8	Liu et al., 2013 [19]
LMD	Heat treatment	Vertical	1036 ± 15	1135 ± 7	10.7 ± 1.2	Liu et al., 2014 [20]
LMD	Heat treatment	Vertical	1067 ± 6	1111 ± 7	12.3 ± 0.6	Liu et al., 2014 [21]
LMD	Heat treatment	Horizontal	957.1 ± 0.007	994.0 ± 0.023	4.7 ± 0.367	Wang et al., 2019 [7]
		Angled (45°)	1045.7 ± 0.011	1101.3 ± 0.010	4.7 ± 0.308	
		Vertical	924.2 ± 0.005	1004.2 ± 0.006	12.0 ± 0.075	
LMD	As-built	Horizontal	-	~1010	~9.9	Liu et al., 2020 [22]
		Angled (45°)	-	~1095	~4.9	
		Vertical	-	~980	~16.3	
EBM	As-built	Horizontal	929 ± 23	1024 ± 21	14 ± 1	Madeja et al., 2020 [23]
		Angled (45°)	994 ± 27	1059 ± 30	8 ± 2	
		Vertical	978 ± 26	1027 ± 31	7 ± 1	
EBM	As-built	Horizontal	-	1042–1230	8–19	Li et al., 2021 [24]
SLM	As-built	Horizontal	801 ± 16	938 ± 4	18.5 ± 1.0	Huang et al., 2021 [25]
SLM	As-built	Vertical	789 ± 1.9	799 ± 2.5	15.9 ± 0.3	
	Heat treatment		1195 ± 10.3	1245 ± 9.9	7.8 ± 0.5	Bai et al., 2021 [27]
	Heat treatment		1295 ± 8.7	1320 ± 7.5	5.5 ± 0.1	
SLM	As-built	Horizontal	853.1 ± 16.2	889.1 ± 18.7	19.4 ± 1.8	Zhang et al., 2022 [28]
	Heat treatment	Horizontal	1235.1 ± 9.4	1264.3 ± 18.7	9.3 ± 0.7	

**Table 2:** Chemical composition of the Ti-55511 powder used in this survey.

Element	Al	Mo	V	Cr	Fe	Si	Zr	C	O	N	Ti
Wt.%	5.20	4.93	5.0	1.10	0.96	0.02	0.01	0.01	0.1389	0.0152	Bal.

**Table 3:** The mechanical properties of as-fabricated Ti-55511 alloys.

Sample	UTS (MPa)	YS (MPa)	E (GPa)	Elongation (%)
X	879.4 ± 19.2	815.0 ± 23.6	59.4 ± 8.6	25.4 ± 1.5
Y	883.3 ± 3.8	833.3 ± 7.6	61.3 ± 2.3	23.7 ± 1.7
Z	874.5 ± 13.1	868.2 ± 7.4	78.5 ± 7.9	17.3 ± 1.6
XY45	885.9 ± 7.3	828.3 ± 10.4	62.7 ± 1.2	26.6 ± 2.6
XZ45	858.7 ± 5.7	831.7 ± 11.6	69.7 ± 1.1	20.9 ± 1.2
YZ45	848.1 ± 12.1	796.7 ± 10.4	64.7 ± 6.2	22.3 ± 1.7

**Table 4:** The mechanical properties of aged Ti-55511 alloys.

Sample	UTS (MPa)	YS (MPa)	E (GPa)	Elongation (%)
H-X	1141.7 ± 11.6	1113.3 ± 15.3	104.4 ± 3.8	14.2 ± 0.9
H-Y	1145.5 ± 14.4	1110.0 ± 10.0	104.4 ± 1.8	13.3 ± 2.0
H-Z	1145.1 ± 26.4	1100.7 ± 26.9	110.7 ± 7.1	15.9 ± 1.7
H-XY45	1132.5 ± 11.5	1093.3 ± 11.6	98.3 ± 2.2	13.4 ± 1.9
H-XZ45	1152.1 ± 21.3	1116.7 ± 15.3	100.5 ± 4.2	15.4 ± 1.4
H-YZ45	1165.6 ± 12.9	1123.3 ± 5.8	99.8 ± 2.6	15.6 ± 0.9

**Table 5:** The structural parameters of the β phase in as-fabricated samples.

Sample	2*θ°* for (110)	*a* (Å)	*FWHM* for (110)	Average Crystallite Size (nm)	Average Strain (10^−3^)	Average Dislocation Density (10^15^ Line/m^2^)	Intensity Ratio *f* (%)
Powder	39.160	3.251	0.201	37.242	2.067	0.684	76.047
X	39.280	3.241	0.382	21.109	3.677	2.164	38.522
Y	39.241	3.244	0.347	20.711	3.709	2.222	71.272
Z	39.240	3.244	0.355	18.004	4.332	3.138	89.574
XY45	39.280	3.241	0.420	20.838	3.752	2.249	33.711
XZ45	39.300	3.239	0.414	18.218	4.237	2.894	71.185
YZ45	39.260	3.243	0.413	18.305	4.317	2.897	88.354

**Table 6:** The structural parameters of the prior-β phase in aged samples.

Sample	2*θ°* for (110)	*a* (Å)	*FWHM* for (110)	Average Crystallite Size (nm)	Average Strain (10^−3^)	Average Dislocation Density (10^15^ Line/m^2^)	Intensity Ratio *f* (%)
H-X	39.619	3.214	0.294	25.950	2.991	1.461	18.268
H-Y	39.599	3.216	0.286	26.131	3.009	1.502	58.364
H-Z	39.560	3.219	0.279	25.848	3.128	1.679	80.540
H-XY45	39.539	3.221	0.309	26.420	3.009	1.487	31.095
H-XZ45	39.580	3.217	0.262	26.663	3.256	1.578	80.734
H-YZ45	39.601	3.216	0.251	25.658	3.026	1.543	81.184

**Table 7:** The structural parameters of the α phase in aged samples.

Sample	2*θ°* for (002)	*c* (Å)	*FWHM* for (101)	*c*/*a*	Average Crystallite Size (nm)	Average Strain (10^−3^)	Average Dislocation Density (10^15^ Line/m^2^)	Intensity Ratio *f* (%)
H-X	38.421	4.682	0.326	1.597	29.670	2.917	1.241	28.387
H-Y	38.480	4.675	0.303	1.596	29.152	2.956	1.265	37.782
H-Z	38.500	4.673	0.29	1.597	28.204	3.064	1.357	46.695
H-XY45	38.441	4.680	0.298	1.598	29.980	2.896	1.232	41.101
H-XZ45	38.420	4.682	0.304	1.598	28.741	3.050	1.318	41.201
H-YZ45	38.480	4.675	0.281	1.597	29.182	3.013	1.328	37.104
